# Potassium Phosphonate Induces Resistance in Sweet Chestnut against Ink Disease Caused by *Phytophthora* Species

**DOI:** 10.3390/pathogens12030365

**Published:** 2023-02-22

**Authors:** Andrea Brandano, Salvatorica Serra, Giles E. St. J. Hardy, Bruno Scanu

**Affiliations:** 1 Department of Agricultural Sciences, University of Sassari, Viale Italia 39A, 07100 Sassari, Italy; 2 Phytophthora Science and Management, Centre for Climate Impacted Terrestrial Ecosystems, Harry Butler Institute, Murdoch University, Perth, WA 6150, Australia; 3 National Biodiversity Future Center S.c.a.r.l. (NBFC), Palazzo Steri, Piazza Marina 61, 90133 Palermo, Italy

**Keywords:** oomycete, ink disease, fungicide, induced resistance, disease control

## Abstract

Ink disease, caused by *Phytophthora* spp., represents a serious threat to sweet chestnuts throughout their distribution area. Among the control strategies, new perspectives have been offered by using potassium phosphonate, which indirectly controls *Phytophthora* diseases by acting on both host physiology and host-pathogen interactions. In this study, we tested *in planta* the effectiveness of trunk injection with K-phosphonate against seven different *Phytophthora* species associated with ink disease. For the two most aggressive species, *P. cinnamomi* and *P*. ×*cambivora*, the treatments were repeated at two different environmental conditions (a mean temperature of 14.5 °C vs. 25 °C) and tree phenology stages. The results obtained in this study demonstrated that K-phosphonate could contain the development of *Phytophthora* infection in phloem tissues. However, its effectiveness varied based on the concentration applied and the *Phytophthora* species tested. A concentration of 280 g/L of K-phosphonate was the most effective, and in some cases, callus formation around the necrotic lesion was detected. Overall, this study broadens the knowledge of endotherapic treatments with K-phosphonate as an effective measure for managing chestnut ink disease. Interestingly, the increase in mean temperature had a positive impact on the development of *P. cinnamomi* lesions on chestnut phloem tissues.

## 1. Introduction

Sweet chestnut (*Castanea sativa* Mill.) forest stands and orchards are part of the traditional and historical European landscape, covering more than 2.5 million ha, and mainly distributed among France and Italy, followed by Spain, Portugal, and Switzerland [1]. Historically, chestnut stands have represented a vital economic resource of the agroforestry ecosystems, particularly in European rural areas, producing timber, firewood, forage, tasty edible fruits, and secondary products such as pasture, hay, mushrooms, tannins, and honey [1,2,3]. In addition, chestnut woodlands play a huge ecological and social role, providing several ecosystem services, such as protection against fire and soil erosion, biodiversity preservation, and conservation of cultural values and recreation, among others [4].

**Figure 1:**
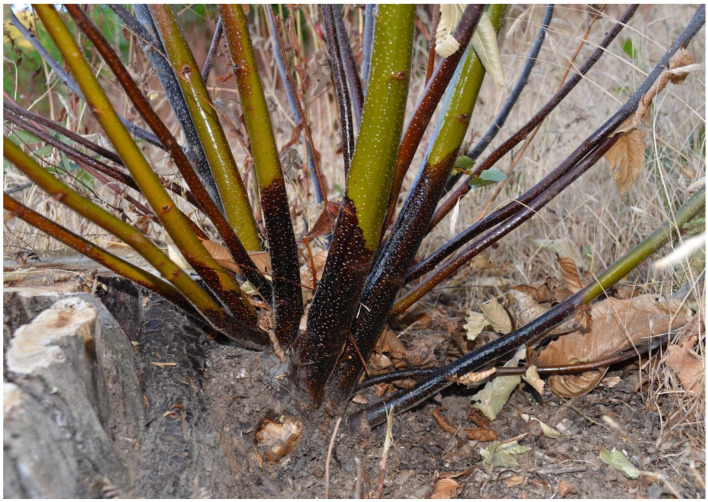
Ink-diseased chestnut stump with new resprouts showing the typical flame-shaped necrotic lesions on the outer bark caused by *P*. ×*cambivora*.

Over the last decades, however, the productivity and sustainability of chestnut stands have been threatened by several factors, including socio-economic changes, climatic reverses, fire, and the introduction and re-emergence of pests and pathogens, which have led to the abandonment of chestnut cultivation in many areas [5,6,7,8,9]. Among diseases, those caused by *Cryphonectria parasitica* (Murr.) Barr (chestnut blight), and *Phytophthora* spp. (ink disease) have negatively impacted chestnut woodlands across Europe [10,11,12]. While the incidence of chestnut blight has been mitigated due to the natural spread of hypovirulence, which has maintained the disease at low severity in most regions [13], ink disease (Figure 1) still represents a serious phytosanitary problem that demands effective means to control [9]. Ink disease management is complex because its incidence is strictly related to climatic and site conditions. These include abundant rainfall and mild temperatures, waterlogging, and poor soil fertility, all exacerbated by anthropogenic activities like soil compaction and disturbance by tillage practices, vehicle movement along roads, movement of contaminated substrates, and planting of infested nursery stock [14,15,16,17,18]. The situation may be even more complex, as in addition to *P. cinnamomi* Rands and *P*. ×*cambivora* (Petri) Buisman, the main species associated with ink disease, other *Phytophthora* species may be recovered from symptomatic chestnut trees. These include *P. cactorum* (Lebert and Cohn) J. Schröt., *P. castanetorum* T. Jung, Horta Jung, Bakonyi and Scanu, *P. cryptogea* Pethybr. and Laff., *P. gonapodyides* (H.E. Petersen) Buisman, *P. megasperma* Drechsler, *P. nicotianae* Breda de Haan, *P. plurivora* T. Jung and T.I. Burgess, *P. pseudosyringae* T. Jung and Delatour, *P. sansomeana* E.M. Hansen and Reeser and *P. syringae* (Kleb.) Kleb. [9,12,19,20,21,22]. Therefore, disease control requires accurate diagnosis and analysis of the site and environmental conditions, other than selecting rigorous control methods.

Several systemic fungicides are active against *Phytophthora*, such as Metalaxyl, Fosetyl-Al, Dimethomorph, and Copper Hydroxide [23,24]. However, their use in forests and natural environments is restricted or prohibited in most European countries. In addition, there are several studies on biocontrol with natural antagonists (both fungi and bacteria), biofumigation with Brassicaceae, or breeding with resistant clones of *C. sativa*. However, their effectiveness in the field is still too low to control the disease [9,25]. Among the main control strategies, treatments with phosphonate (formerly named phosphite) are the most common and successful methods for controlling *Phytophthora* on woody trees in horticulture and natural ecosystems [26,27,28,29,30,31,32,33]. Phosphonates (phosphoric acid) are fully systemic fungicides, i.e., they are xylem- and phloem-translocated with both downward and upward movement in the host [34]. The precise mechanism of action of phosphonates is complex and not fully understood. However, both direct effects on pathogen growth and sporulation and indirect effects by stimulating host plant defenses have been demonstrated by several studies [35,36,37,38]. While phosphonate enhances fine root formation in treated woody trees, it does not seem to alter the abundance and composition of edaphic, floristic, and endophytic bacteria in trees or the balance between different microorganisms, including *Phytophthora* species [33,39], although this needs further study. Phosphonates can be applied by foliar spray, soil drenches, trunk painting, and injection [40,41]. Several commercial phosphonate formulations are used on woody trees, including potassium, sodium, and ammonium phosphonate [34,42]. Stem injection with K-phosphonate (K_2_HPO_3_) has been previously tested to control ink disease on sweet chestnut [43,44,45]. These studies were focused on the efficacy against either *P*. ×*cambivora* or *P. cinnamomi*, while very little is known about the effect of endotherapic treatments against other *Phytophthora* species associated with ink disease and the influence of the temperature in field experiments. Therefore, the present study was undertaken to (i) identify *Phytophthora* species previously obtained from ink-diseased chestnut trees; (ii) test the effectiveness of K-phosphonate against several *Phytophthora* species associated with ink disease; (iii) detect the most appropriate K-phosphonate concentration for stem injection and its phytotoxicity threshold; and (iv) evaluate whether environmental temperature has any effects on control treatments and the development of *Phytophthora* infection.

## 2. Materials and Methods

### 2.1. Collection and Identification of Phytophthora Isolates

Isolates of *Phytophthora* were sourced from the culture collection of the University of Sassari, Italy, and directly from ink-diseased chestnut trees in Sardinia (Table 1). Isolations were made by direct isolation from the typical flame-shaped necrotic lesions on phloem tissues and/or by baiting the rhizosphere soil samples of symptomatic trees [21]. The modified Synthetic Mucor Agar (SMA), a selective medium for *Phytophthora*, was used in both isolation methods [46]. Any growing colonies developed on SMA were subcultured and stored on carrot agar [47] (CA) at 20 °C. Isolates obtained were first grouped based on morphological analyses, and then representative morphotypes (8) were subjected to molecular analyses as described by Scanu and Webber [48]. DNA extraction, amplification, purification, sequencing, and analysis of the ITS sequences were performed as described by Scanu et al. [49]. All sequences were deposited at GenBank (http://www.ncbi.nlm.nih.gov/), and accession numbers are given in Table 1, together with all isolates’ information. All species were obtained from ink-diseased trees in chestnut stands in Sardinia, although two isolates (*P. cinnamomi* and *P*. ×*cambivora*) were derived from declining *Q. suber* trees (Table 1). Stock cultures were maintained on CA tubes at 12 °C at the culture collection of the University of Sassari. All isolates were passaged (inoculated and re-isolated) through host plant material prior to their use in experiments to ensure their pathogenicity was not lost during culture storage.

### 2.2. Study Site and Experimental Design

**Figure 2:**
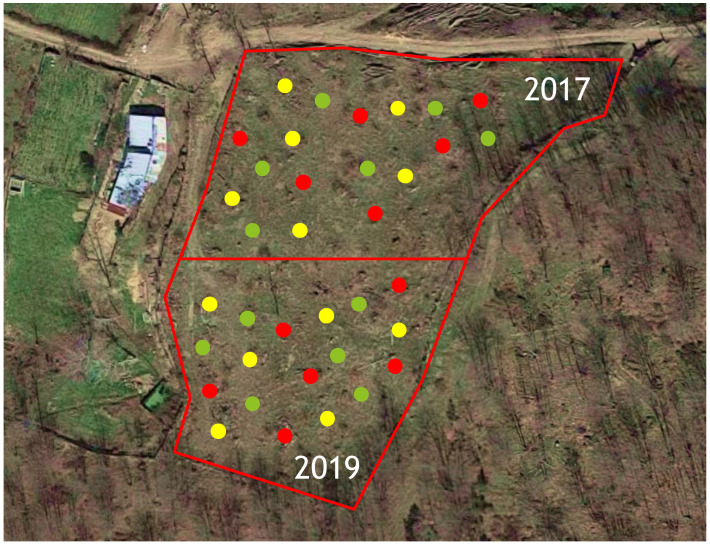
Experimental site with 36 coppices in which the stems were injected with 0 g/L (green dots), 140 g/L (yellow dots) and 280 g/L (red dots) of K-phosphonate in 2017 and 2019.

The experimental trials were conducted in two neighboring sweet chestnut stands located along a mountain slope in the Gennargentu area of Sardinia, Italy (40°01′06″ N, 9°14′40″ E; 1080 m a.s.l.). Both stands consisted of newly cut coppices with 3-year-old resprouts (in the following called “stems”), which were considered healthy as they did not show any specific symptoms of chestnut blight or ink disease. In experiment 1, in each coppice, twenty-one stems were selected, while in experiments 2 and 3, twelve stems were chosen, with each having at least 7 cm in diameter at the base. These were marked for K-phosphonate treatment (the main plot factor) and arranged in a split-plot design with six replicate blocks (coppices). A total of 60 coppices were randomly selected: 24 for experiment 1, and 18 each for experiments 2 and 3. Seven *Phytophthora* species, artificially inoculated in the stems (below), were randomly assigned as a subplot within each of the coppice main plots. The experiments were undertaken during September 2016 (experiment 1) in one chestnut stand and during August 2017 and May 2019 (experiments 2 and 3) in a second stand (Figure 2).

### 2.3. Stem Injection of K-Phosphonate

**Figure 3:**
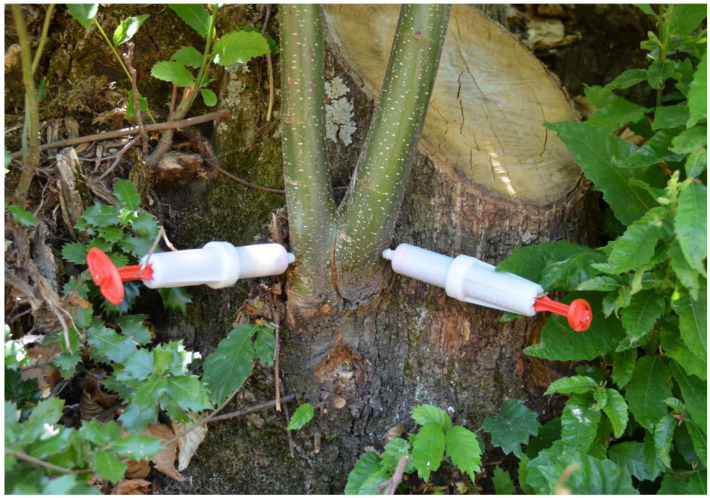
Chestnut resprouts are injected at the base of the stem with K-phosponate using Chemjet^®^ Tree syringes.

The selected chestnut stems were injected with aqueous phosphonate solutions made from a 70% commercial formulation (Kalex^TM^, Alba Milagro, Parabiago, Italy), containing 700 g/L of potassium phosphonate (K_2_HPO_3_), adjusted to pH 4–5. Three different concentrations of K-phosphonate were tested, including 10% (70 g/L), 20% (140 g/L), and 40% (280 g/L) diluted with deionized water. The 10% concentration was tested only in the first experiment. K-phosphonate was injected at the base of each selected stem. A hole was drilled at the bottom of the stems through the outer bark layer into the sapwood with a 5.5 mm drill bit. Then the K-phosphonate solutions were injected using 20-mL spring-loaded tree syringes (Chemjet^®^ Tree Injector, Chemjet Europe and Middle East, UK), one per stem, that lock tightly into the stem (Figure 3). For control treatments, the same procedure was followed, but 20 mL of sterile water was injected instead of K-phosphonate. Stems in the same coppice received the same K-phosphonate concentration, and there were six replicate coppices for each K-phosphonate concentration. One syringe per stem was used, and the time for uptake of the solution varied from 5 to 30 min. After injection, the hole was sealed with healing resin (Arbokol, Kollant, Vigonovo (VE), Italy) for pruning wounds to avoid any fungal infection. The foliage and stems of treated coppice were monitored for phytotoxicity for up to 2 days. Those individuals showing symptoms like pale brown discoloration of leaf margins and tip necrosis were excluded from the trials.

### 2.4. Stem Inoculation of Phytophthora

Two days after K-phosphonate treatments, chestnut stems were artificially inoculated with isolates of different *Phytophthora* species (Table 1), following the experimental design. While the first experiment included seven different *Phytophthora* spp., *P. castanetorum*, *P. cinnamomi*, *P. gonapodyides*, *P. megasperma*, *P. plurivora*, *P. pseudosyringae*, and *P*. ×*cambivora*, the second and third experiments focused only on *P*. ×*cambivora* and *P. cinnamomi.* Stem inoculations were made between 80 and 120 cm above the collar following the method used by Scanu and Webber [48]. After sterilizing the bark with 70% ethanol, a 7-mm-diameter hole was punched through the bark to the wood surface with a steel cork borer. The same-sized plug was taken from the edge of a *Phytophthora* colony actively growing on a 90-mm Petri dish of CA and used as inoculum by inserting it into the hole, replacing the bark plug. Moist cotton wool was placed over the wounds, covered with a 5 × 5 cm piece of aluminium foil, and sealed with an adhesive PVC tape. In the first experiment, made in September 2016, only one isolate per *Phytophthora* species was used, while in the second and third experiments, made in August 2017 and May 2019, respectively, two isolates of the most aggressive and main *Phytophthora* species associated with ink disease, *P*. ×*cambivora*, and *P. cinnamomi* [9], were used (Table 1). Within each chestnut coppice, each isolate was inoculated twice into individual stems, approximately 50 cm apart, to give a total of 12 inoculated stems (three replicate stems for each *Phytophthora* isolate). After 35 days, the inoculated stems were harvested and transferred to the laboratory to analyze the developing necrotic lesions. The periderm of each stem was destructively removed with a drawknife to expose the phloem. Each lesion was outlined and recorded on tracing paper, and then scanned on an Epson Perfection V30 photo scanner. The lesion area was calculated using APS Assess 2.0 (image analysis software for disease quantification; The American Phytopathological Society, St. Paul, MN, USA). Re-isolation of all the inoculated *Phytophthora* species onto SMA was attempted from the lesion margins. The cultures obtained were compared with the isolates used for the inoculation.

During the experiments, the temperatures were recorded with two data loggers (EL-USB-2-LCD Data logger, Lascar Electronics, Erie, PA, USA), which were placed in two different coppices. The temperature data, scanned every 15 min, were downloaded and analyzed with the software EasyLog Graph Version 7.4.0.0 (Lascar Electronic, Erie, PA, USA).

### 2.5. Statistical Analyses

Statistical analyses were performed using the software R and RStudio (R Core Team, 2017). R: A language and environment for statistical computing (R Foundation for Statistical Computing, Vienna, Austria). Linear mixed models (LMMs), which are strongly recommended when unbalanced samples occur, were used and fitted using Restricted Maximum Likelihood estimation (REML) [50]. Models were fitted separately for each pathogen using the *lmer* function from the “lme4” package in R [51]. Before the analysis, all data were explored for heteroscedasticity and normal distribution. In LMMs, the K-phosphonate treatment (i.e., control, 140 g/L and 280 g/L) was considered a fixed factor, whereas the stem nested within each selected coppice was selected as a random factor to account for the split-plot experimental design. Statistical differences among mean values of the lesion areas for each *Phytophthora* species were assessed using Analysis of Variance (ANOVA), followed by the Fisher’s protected least significant difference (LSD) test and Tukey HSD test (Honestly Significative Difference). Differences in mean values with *p* ≤ 0.05 were considered significant.

## 3. Results

### 3.1. Isolation and Identification of Phytophthora Species

Details of the *Phytophthora* species used in this study are provided in Table 1. Only *P.* ×*cambivora* was isolated from necrotic lesions detected on phloem tissues on chestnut trees, while *P. castanetorum*, *P. gonapodyides*, *P. megasperma*, and *P. pseudosyringae* were obtained from rhizosphere soil samples of symptomatic mature trees of sweet chestnut using the baiting method. An isolate of *P. cinnamomi* and *P. plurivora* were detected in chestnut seedlings in a nursery and newly planted chestnut saplings, respectively. While one isolate each of *P. cinnamomi* and *P.* ×*cambivora* were obtained from the rhizosphere soil of declining *Q. suber* trees. All morphological characters and ITS sequences matched those of the corresponding *Phytophthora* species (Table 1).

### 3.2. Experiment 1

**Figure 4:**
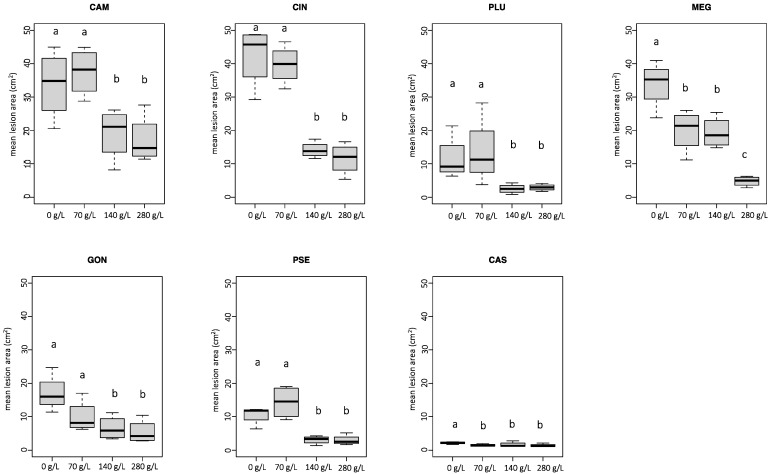
Mean lesion area (cm^2^) caused by *Phytophthora* ×*cambivora* (CAM), *P. cinnamomi* (CIN), *P. plurivora* (PLU), *P. megasperma* (MEG), *P. gonapodyides* (GON), *P. pseudosyringae* (PSE), and *P. castanetorum* (CAS) on chestnut stems treated with four different concentrations of K_2_HPO_3_ (0 g/L, 70 g/L, 140 g/L, and 280 g/L). Bars represent the standard error. Box plots with the same letters did not show statistically significant differences on the HSD Tukey test for *p* ≤ 0.05.

On all chestnut stems inoculated with *Phytophthora*, a characteristic necrotic lesion developed. The mean lesions caused by all *Phytophthora* species on treated stems with 140 g/L and 280 g/L of K-phosphonate concentrations were consistently smaller than those that developed on the control inoculations (Figure 4). In contrast, lesion areas did not differ significantly between stems injected with 70 g/L and controls, with the exception of *P. megasperma* and *P. castanetorum*. In some cases (*P. plurivora*, *P. pseudosyringae*, and *P*. ×*cambivora*), were even larger on average than controls (Figure 4). For *P. megasperma*, the mean lesion developed on stems treated with 280 g/L of K-phosphonate concentration was significantly different from that on stems treated with 70 g/L and 140 g/L concentrations. *Phytophthora* ×*cambivora* and *P. cinnamomi* were shown to be the most aggressive species in colonizing phloem tissues on untreated controls, with a mean lesion area of 33.8 cm^2^ and 42.4 cm^2^, respectively. Interestingly, *P. megasperma* showed to be as much aggressive as *P.* ×*cambivora* and *P. cinnamomi*. No phytotoxicity was observed in stems injected with K-phosphonate.

There was 100% pathogen re-isolation from lesions developed on both K-phosphonate-treated and untreated stems, except for *P. castanetorum,* which showed 40% re-isolation from around the inoculation points. During the trial, the temperature ranged from a minimum of 1.5 °C to a maximum of 30 °C, with a mean temperature of 13.3 °C.

### 3.3. Experiment 2

**Figure 5:**
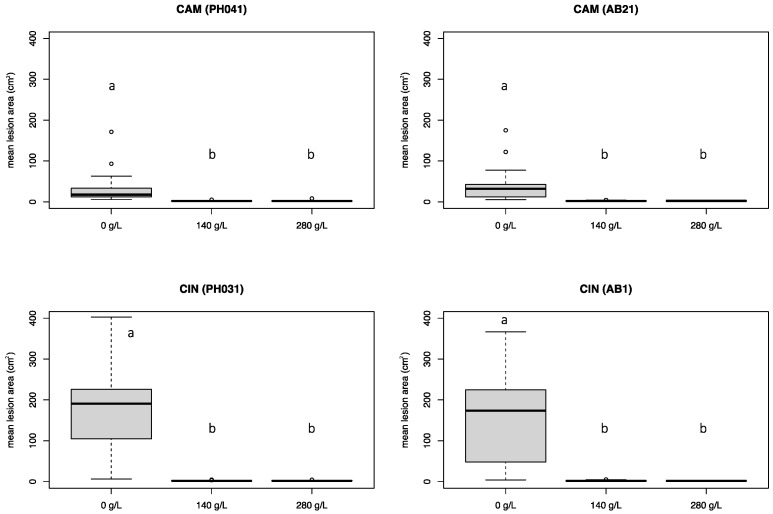
Mean lesion area (cm^2^) caused by *Phytophthora* ×*cambivora* (PH041, AB21) and *P. cinnamomi* (PH031, AB1) on chestnut stems treated with three different concentrations of K_2_HPO_3_ (0 g/L, 140 g/L, and 280 g/L). Bars represent the standard error. Box plots with the same letters did not show statistically significant differences on the HSD Tukey test for *p* ≤ 0.05.

**Figure 6:**
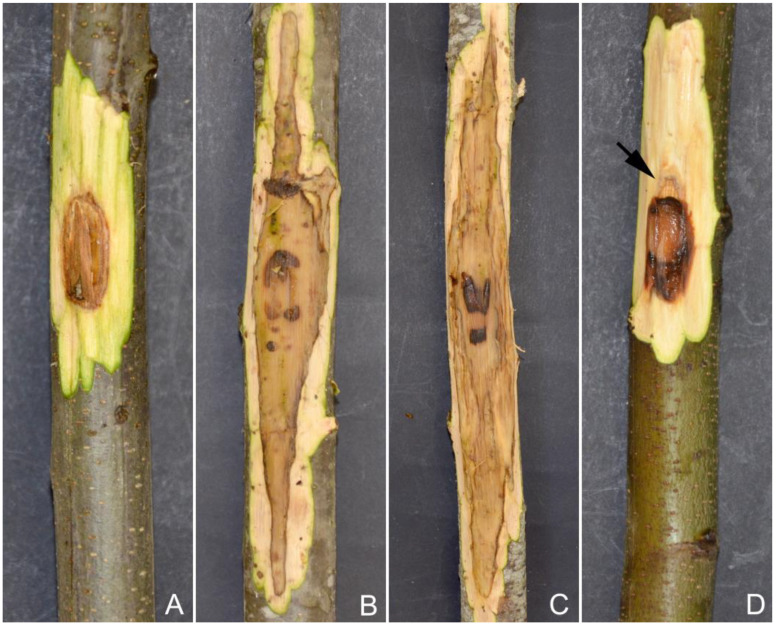
Lesion developed on treated and untreated chestnut stems: a contained lesion area caused by *Phytophthora* ×*cambivora* on a stem previously injected with K-phosphonate (280 g/L) (**A**), a lesion caused by *P.* ×*cambivora* and *P. cinnamomi* in control treatments (**B**,**C**), a contained lesioned area caused by *P. cinnamomi* on a stem previously injected with K-phosphonate (280 g/L) showing callus formation (arrow) around the inoculation point (**D**).

Based on the results obtained in the first experiment, two isolates of each of the two most aggressive *Phytophthora* species (*P.* ×*cambivora* and *P. cinnamomi*) (Table 1) and the two most effective K-phosphonate concentrations (140 g/L and 280 g/L) were used in experiment 2. Both K-phosphonate concentrations significantly (*p* < 0.05) reduced the development of necrotic lesions caused by all *Phytophthora* isolates (Figure 5 and Figure 6). No statistical differences were found between the two K-phosphonate concentrations. *Phytophthora cinnamomi* caused extensive necrotic lesions on untreated stems, with a mean lesion area of 171.7 cm^2^, which was, on average, almost five times larger than those caused by *P.* ×*cambivora* (mean value 34.5 cm^2^). Some phytotoxicity was observed in stems injected with 280 g/L of K-phosphonate.

All *Phytophthora* isolates were readily re-isolated from the necrotic lesions visible in the phloem tissues of both treated and untreated stems. In some cases, stems treated with 280 g/L of K-phosphonate only developed limited discoloration around the inoculation point and did not yield any *Phytophthora* when isolation was attempted. The stems treated with the highest concentration showed callus formation, which tended to confine the necrotic lesion, particularly the upward development (Figure 6D). During the experiment, the recorded temperatures ranged from a minimum of 12 °C to a maximum of 37 °C, with a mean temperature of 25 °C.

### 3.4. Experiment 3

**Figure 7:**
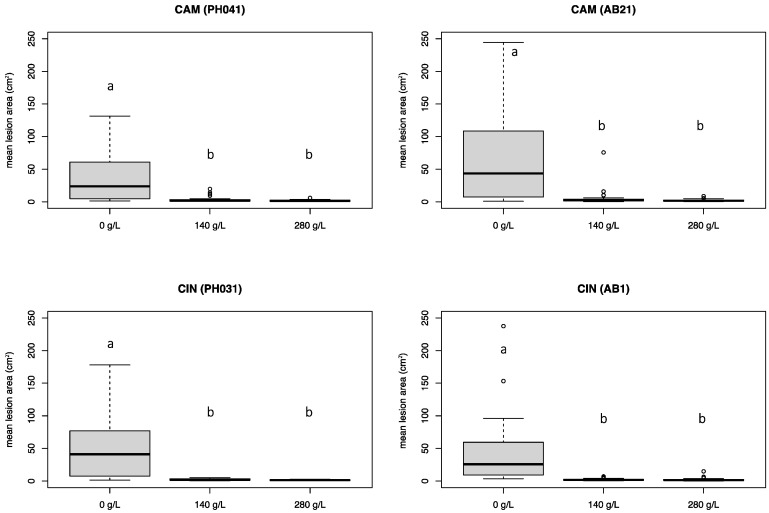
Mean lesion area (cm^2^) caused by *Phytophthora* ×*cambivora* (PH041, AB21) and *P. cinnamomi* (PH031, AB1) on chestnut stems treated with three different concentrations of K_2_HPO_3_ (0 g/L, 140 g/L, and 280 g/L). Bars represent the standard error. Box plots with the same letters did not show statistically significant differences on the HSD Tukey test for *p* ≤ 0.05.

Similarly to experiment 2, both K-phosphonate concentrations were able to significantly reduce (*p* < 0.05) the development of necrotic lesions caused by both *P.* ×*cambivora* and *P. cinnamomi* isolates (Figure 7). The 280 g/L concentration of K-phosphonate was more effective than the 140 g/L concentration in reducing the growth of *P. cinnamomi* (96.2% vs. 95.3%) and *P.* ×*cambivora* (96.3% vs. 91.3%) on injected stems, compared with untreated stems. However, no statistical differences were found between necrotic lesions developed on stems injected with both concentrations, demonstrating their effectiveness. On control stems, *P.* ×*cambivora* was equally aggressive and caused lesions similar in size to those produced by *P. cinnamomi*, which were, on average, almost 50 cm^2^ in size. Phytotoxicity was not detected in any of the treated chestnut stems.

All *Phytophthora* isolates were readily re-isolated from the necrotic lesions visible in the phloem tissues of both treated and untreated stems. During the trial, the temperature ranged from a minimum of 1 °C to a maximum of 32 °C, with a mean temperature of 14.5 °C.

## 4. Discussion

The management of *Phytophthora* diseases in forest ecosystems still represents a challenge since scarce field experimental data about control strategies is available. This study provides further evidence of the efficacy of phosphonates in suppressing *Phytophthora* infection in woody trees [34,35,36,37,38,39,40,41,42,43,44,45]. Trunk injection with K-phosphonate significantly reduced the development of necrotic lesions on phloem tissues of chestnut trees artificially inoculated with a range of *Phytophthora* species associated with ink disease. 

Phosphonate has been widely used in agricultural, forest, and natural settings to directly control *Phytophthora* by inhibiting its growth and sporulation and especially to stimulate host defense responses [23,29]. The direct effect of K-phosphonate *in planta* against *Phytophthora* is difficult to demonstrate [52]. In contrast, the containment of *Phytophthora* infection through a stimulated coordinated response following phosphonate injection was shown in this study by the formation of callus tissue around the margins of almost all lesions (Figure 6). in contrast no callus was observed in untreated stems, which is consistent with previous studies [38,53]. K-phosphonate may increase the percentage of healing attributable to its dual ability to slow the growth of the pathogen while enhancing the growth of the host plant and compartmentalizing lesions caused by *Phytophthora* [23]. Similar compartmentalization of necrotic lesions has been noted for *Banksia brownii* infected by *P. cinnamomi* following treatment with potassium phosphonate [51]. A transcriptomics analysis would be helpful to determine whether the phosphonate treatments are linked with the expression of microbial genes that might inhibit *Phytophthora*, as demonstrated previously [38]. 

K-phosphonate can be applied in different ways to protect chestnut trees, including soil drenches, foliar sprays, trunk paints, and injection [29,44,54]. However, trunk injection increases the efficiency of K-phosphonate to contain disease development as it allows the application of much higher concentrations than foliar and stem sprays. Indeed in the first experiment, the lowest concentration of K-phosphonate (70 g/L) did not significantly reduce the development of necrotic lesions for almost all *Phytophthora* species tested, compared to the untreated controls. Furthermore, once injected, K-phosphonate is rapidly translocated through the xylem and phloem up and down the tree [55]. This was also shown in this study, in the second experiment, when symptoms of phytotoxicity such as yellowing and browning were observed on leaves after 48 h from the treatment, indicating the systemic action of K-phosphonate [29]. Although it was only associated with small-sized treated stems and only detected in individuals treated with a 280 g/L concentration, phytotoxicity remains a concern. It must be balanced against the improvement in the health of most trees treated with K-phosphonate [55]. Trunk injection may also be considered invasive for the tree since the drill wounds can allow the entry of pathogenic bacteria or other fungi, i.e., *C. parasitica* on sweet chestnut. However, in all experiments, treated trees remained healthy and the wounds were successfully compartmentalized. Another disadvantage is that the absorption of K-phosphonate can vary among the physiological conditions of the treated trees and depend on climate variables, which can influence a plant’s transpiration rate [44]. Tree phenology may be another factor that can regulate the efficiency of the translocation of phosphonate to the roots [56,57]. However, the effectiveness of the treatments did not change in our experiments. For deciduous trees in temperate climates, summer injections are ideal for upward movement and should be avoided in late autumn and winter [58]. Most registered phosphonate products on woody trees usually recommend applying two annual trunk injections, which seem unsustainable in extensive forests due to the labor-intensive and costly nature of the injections. Previous studies reported that the effectiveness of K-phosphonate can last from 2 to 6 years, depending on the host plant and *Phytophthora* species involved in the pathosystem [29]. Further research is needed to determine how long phosphonate remains active in treated chestnut trees.

Despite the effectiveness of K-phosphonate in controlling *Phytophthora* diseases and its low environmental impact, its use is regulated differently worldwide. K-phosphonate is considered a systemic fungicide in Australia, the USA, and South Africa, where it is widely used in agriculture, agroforestry, and forestry [23,29]. In most European countries, including Italy, K-phosphonate has until now been regulated by Reg. CE n. 369/2013 and registered as fertilizers [59], thus with almost no restrictions. Since scientific evidence shows that phosphonates have no nutritional role in the plant to justify their use as fertilizer, recently, the European Directive EU 1009/2019 has restricted their use, with a ban on their use in both conventional and organic agriculture. Therefore, any new formulation based on phosphonate will be registered as a pesticide/fungicide on target crops/plants [60]. Currently in Italy K-phosphonate is registered on seven fruit bearing trees, tomato and grape. Therefore, at least in Europe, using K-phosphonate to control chestnut ink disease will depend on country-based regulations. In addition, treatments of K-phosphonate must also consider the Maximum Residue Limit (MRL) recently issued by EU Regulation 2021/1807, which increases the limit for chestnut fruits to 1500 mg/kg. Considering the efficacy of K-phosphonate against other important pathogens on sweet chestnut, such as *Gnomoniopsis castanea* [60], further research focused on residue values after treatments is needed. Previous studies reported lower values than those issued by the EU regulation 2021/1807 [59,60].

Because of these new limitations on the use of phosphonates, valid alternative treatments must be researched in the future. Silicate-based mulch could be a valuable alternative to phosphonates, especially where there might be evidence of resistant isolates [61,62]. Recently, Ca chelate is another potential product that can be utilized to stimulate plant defense responses against plant pathogens, particularly *P. cinnamomi* [63]. Green pesticides like those based on cinnamate anion and bioactive metabolites produced by fungi have shown a strong inhibition rate comparable to some fungicides against *Phytophthora* spp., including *P. cinnamomi* and *P*. ×*cambivora* [64,65]. Biocontrol beneficial microorganisms such as Proteobacteria (e.g., *Bacillus* spp.), Actinobacteria (e.g., *Streptomyces* spp.), Gammaproteobacteria (e.g., the fluorescent *Pseudomonas*), and fungi (e.g., non-pathogenic *Fusarium* spp. and *Trichoderma* spp.) have shown promising results against several foliar and soilborne diseases [66,67]. The antagonistic role of these microorganisms against *Phytophthora* deserves further investigation [68].

An additional outcome of this study is that *P*. ×*cambivora* and *P. cinnamomi* were confirmed to be the most aggressive *Phytophthora* species in colonizing phloem tissues on sweet chestnut [9,11,12]. Lesion development by *P. cinnamomi* may have been due to the higher temperatures during experiment 2, where, with a mean temperature of 25 °C, *P. cinnamomi* caused extensive lesions (av. 2 cm length per day) on untreated stems. This is consistent with the optimum temperature for pathogen growth in vitro, which is shown to be around 27 °C [49]. In a scenario of climate change, with an expected rise in the mean temperature in the following decades [69], the spread and impact of *P. cinnamomi* on chestnut stands will most likely increase in Mediterranean areas [70,71]. In contrast, the aggressiveness of *P*. ×*cambivora* was not affected by the increase in mean temperature during the trials. Although less aggressive, some *Phytophthora* species caused significant necrotic lesions on untreated stems, such as *P. plurivora* and *P. megasperma*. For the latter, only the highest K-phosphonate concentration of 280 g/L significantly reduced the lesion progress, suggesting some tolerance in this species. Development of resistance to phosphonate after prolonged exposure has been observed in some *Phytophthora* species [60], including *P. crassamura*, a close relative of *P. megasperma* [72]. This suggests that caution should be taken when relying on phosphonate as the only means to control *Phytophthora* diseases [73]. *Phytophthora castanetorum*, *P. gonapodyides*, and *P. pseudosyringae* were shown to be weak colonizers of phloem tissues. However, these species are known for their prevalent soilborne lifestyle; therefore, their involvement in chestnut ink disease may be more related to root infection [9]. Due to the complexity of ink disease, stringent hygiene protocols should be followed to prevent its spread before, during, and after treatment operations. Ink disease incidence is also strictly related to climatic and site conditions and human activities [15,18]. Therefore, any possible strategy should be part of an integrated management program to mitigate chestnut ink disease [74,75].

## 5. Conclusions

Overall, this study broadens the knowledge of using K-phosphonate to control chestnut ink disease caused by *Phytophthora* species [43,44,45]. Although field experiments are often time-consuming and expensive, this study provides a reliable and practical control treatment useful for sweet chestnut growers who have ink disease. The efficacy of trunk injection varied based on the concentration applied and the *Phytophthora* species tested, while it was not affected by tree phenology or environmental temperature during the treatments [44,54,55]. Although K-phosphonate has a low environmental impact, its availability on the market can be locally uncertain depending on the differences in categorization and authorization for its use [57,58].

This work also highlights the potential for some species to become serious pathogens on sweet chestnut in Mediterranean regions under the current climate change scenario. Climate change, in particular a rise in mean temperatures, extreme precipitation regimes, and severe droughts, could intensify ink disease incidences and further destabilize chestnut stands [71,76]. In the absence of alternative methods to control *Phytophthora* disease in forests, trunk injection with K-phosphonate remains a valid solution to mitigate the emergence of *P. cinnamomi* and, overall, reduce the impact of ink disease in chestnut stands.

## Figures and Tables

**Table 1:** Details of *Phytophthora* isolates associated with ink disease and used in this study for stem inoculation.

*Phytophthora* sp.	Isolate Codes	Year	Host	Substrate	GenBank Accessions	Experiment
*P. castanetorum*	P14	2014	*Castanea sativa*	Rhizosphere soil	MF036189	1
*P. cinnamomi*	PH031	2008	*Castanea sativa*	Root	OP918117	1, 2, 3
*P. cinnamomi*	AB1	2016	*Quercus suber*	Rhizosphere soil	OP918118	2, 3
*P. gonapodyides*	PH038	2009	*Castanea sativa*	Rhizosphere soil	OQ176729	1
*P. megasperma*	PH178	2010	*Castanea sativa*	Rhizosphere soil	KP863491	1
*P. plurivora*	PH089	2010	*Castanea sativa*	Rhizosphere soil	OP918116	1
*P. pseudosyringae*	PH043	2009	*Castanea sativa*	Root	OP918115	1
*P. ×cambivora*	PH041	2009	*Castanea sativa*	Collar	OP918113	1, 2, 3
*P. ×cambivora*	AB21	2016	*Quercus suber*	Rhizosphere soil	OP918114	2, 3

## Data Availability

Not applicable.
